# Associations of Cough Prevalence with Ambient Polycyclic Aromatic Hydrocarbons, Nitrogen and Sulphur Dioxide: A Longitudinal Study

**DOI:** 10.3390/ijerph13080800

**Published:** 2016-08-09

**Authors:** Enoch Olando Anyenda, Tomomi Higashi, Yasuhiro Kambayashi, Thao Thi Thu Nguyen, Yoshimasa Michigami, Masaki Fujimura, Johsuke Hara, Hiromasa Tsujiguchi, Masami Kitaoka, Hiroki Asakura, Daisuke Hori, Yohei Yamada, Koichiro Hayashi, Kazuichi Hayakawa, Hiroyuki Nakamura

**Affiliations:** 1 Department of Environmental and Preventive Medicine, Graduate School of Medical Sciences, Kanazawa University, 13-1 Takara-machi, Kanazawa 920-8640, Japan; enolany@yahoo.com (E.O.A.); ykamba@med.kanazawa-u.ac.jp (Y.K.); toi_fs@yahoo.com (T.T.T.N.); t-hiromasa@med.kanazawa-u.ac.jp (H.T.); m-ymzk16@stu.kanazawa-u.ac.jp (M.K.); heros@stu.kanazawa-u.ac.jp (H.A.); hori_d@mbr.nifty.com (D.H.); yamada503597@gmail.com (Y.Y.); orihciok1003@gmail.com (K.H.); 2 Department of Hygiene, Graduate School of Medical Sciences, Kanazawa University, 13-1 Takara-machi, Kanazawa 920-8640, Japan; tomotomo@med.kanazawa-u.ac.jp; 3 Environment Preservation Center, Kanazawa University, Kakuma-machi, Kanazawa 920-1192, Japan; mitigami@t.kanazawa-u.ac.jp; 4 Respiratory Medicine, Cellular Transplantation Biology, Graduate School of Medical Sciences, Kanazawa University, 13-1 Takara-machi, Kanazawa 920-8640, Japan; fujimura@nanao.hosp.go.jp (M.F.); hara0728@gmail.com (J.H.); 5 Respiratory Medicine, National Hospital Organization Nanao Hospital, 3-1 Mattou-machi Yabe, Nanao, Ishikawa 926-8531, Japan; 6 Institute of Medical, Pharmaceutical and Health Sciences, Faculty of Pharmacy, Graduate School of Natural Science and Technology, Kanazawa University, Kakuma-machi, Kanazawa, Ishikawa 920-1192, Japan; hayakawa@p.kanazawa-u.ac.jp

**Keywords:** polycyclic aromatic hydrocarbon, adult patients, asthma, generalized estimating equation, total suspended particles, chronic cough

## Abstract

Information on potential cough triggers including environmental irritants is vital for successful management of chronic cough in patients. We investigated the relationship between ambient levels of particulate polycyclic aromatic hydrocarbons (PAH), nitrogen dioxide (NO_2_) and sulphur dioxide (SO_2_) exposures with cough prevalence. Eighty-three adult patients, who had been physician diagnosed with at least asthma, cough variant asthma and/or atopic cough, were divided into asthma and non-asthma groups. They recorded daily cough symptoms during 4 January–30 June 2011 study period while daily samples of total suspended particles were simultaneously collected by use of glass fiber filters and the particulate PAH content determined by high performance liquid chromatography coupled with a fluorescence detector. Ambient concentrations of NO_2_ and SO_2_ were obtained from a local monitoring site. Logistic regression models using generalized estimating equations were used to determine population-averaged estimates of association between cough prevalence and ambient pollutant exposures for the two groups. Fully adjusted odds ratios from single pollutant models were 1.083 (95% confidence interval (CI): 1.029, 1.140) and 1.097 (95% CI: 1.016, 1.185) per 0.57 ng/m^3^ for lag2 PAH exposure, while only for asthma group had significant associations with NO_2_ and SO_2_ exposures for both lag2 and lag02. Similar associations were observed in multipollutant models. This finding suggests that ambient PAH, NO_2_, and SO_2_ exposure even at low levels is related to cough prevalence in adult chronic cough patients and may be considered as aggravating factor during clinical management of the condition.

## 1. Introduction

The estimated prevalence of chronic cough is said to be affecting close to 10% of the general population [1,2,3]. It is one of the reasons for seeking medical consultation with primary care or respiratory physician, and failure to satisfactorily control it may lead to decreased quality of life in some patients. In clinical settings, chronic cough is often associated with asthma, gastroesophageal reflux disease (GERD) and rhinosinusitis. Furthermore, it is documented that approximately 40% of the referred cases for specialist evaluation may have unknown causes [4]. Heightened cough reflex triggered even by low levels of mechanical, thermal or chemical exposure is a key abnormality in chronic cough [5]. However, poor knowledge of the mechanisms that trigger cough response in experimental animal models and in humans is cited as one of the reasons for the current lack of satisfactory therapies [6]. Although patients with chronic cough report a wide range of triggers, it is important to know the factors that might initiate as well as sustain the condition. Such information will be invaluable in management of patients, alleviating the burden that is associated with the condition.

A recently published review on epidemiological characteristics in parts of Asia revealed that exposure to environmental pollutants and irritants, old age, female gender and comorbidities are important determinants of chronic cough among adults [7]. Exposure to environmental irritants, including ambient pollutants known to trigger cough both in children and adults, has been reviewed [8,9,10]. In addition, there is evidence from cross-sectional studies that have shown the association in adults [11,12]. Although longitudinal studies have also been performed in Europe and North America [13,14] with some inconsistent results, few have specifically studied ambient polycyclic aromatic hydrocarbons (PAH) among adults [15,16]. Likewise, there have been few reported studies in Japan [17,18] which have examined ambient particulate PAH exposure alongside other pollutants. PAHs are a group of organic chemical compounds widely spread in the environment as a result of incomplete combustion and are closely linked to diesel exhaust particles (DEP), PM_2.5_ and PM_10_. Recent data suggests stimulation of Transient Receptor Potential Ankyrin 1 (TRPA1) plays an important role in mediating cough response, and several environmental irritants that are present in air pollution, vehicle exhaust, and cigarette smoke have been demonstrated to be potential agonists of the receptor [19,20,21].

Asthma is a common clinical condition associated with chronic cough, and its prevalence among Japanese adults is said to have increased in the past decade [22]. Additionally, cases of cough variant asthma (CVA), considered a precursor of asthma, and atopic cough (AC) have risen in the recent years [17]. Both of these conditions are known to be major causes of non-productive cough among adult patients in Japan [23]. While the mechanisms of cough sensitivity in these sub-groups of adult patients remain unclear, it may be hypothesized such patients might have increased cough responses that are linked to activation of TRPA1 following exposure to ambient pollutants. It is therefore necessary to assess whether ambient particulate PAHs by themselves are directly associated with cough symptom or act together with other criteria pollutants. The gained information might be useful in clinical management of the condition in this sub-population of patients.

We performed longitudinal analyses on adult patients who were physician-diagnosed with at least asthma, CVA and/or AC, with the aim of assessing whether daily ambient particulate PAH, nitrogen dioxide (NO_2_) or sulphur dioxide (SO_2_) have an impact on cough prevalence. Also, we examined whether there are differences in the associations between sub-groups of patient based on physician diagnoses, gender and age group. Daily cough symptoms were collected by use of diaries while daily ambient pollutants of interest were simultaneously monitored for a period of six months.

## 2. Methods

### 2.1. Participants

Data used were from longitudinal study performed from 4 January to 30 June 2011 on 99 outpatients receiving treatment at Kanazawa University Hospital, Ishikawa Prefecture, Japan. All were adult patients aged over 20 years, physician-diagnosed to have at least asthma, CVA and/or AC during the study period. The composition included asthma (56%), asthma and AC (6%), CVA (9%), AC (18%), and both CVA and AC (11%). In this study we divided them into asthma and non-asthma (CVA and AC) groups. Sixteen patients were excluded from the current analyses due to being current smokers (5%), lack of information on smoking status (3%), no cough information for entire study period (3%), or diagnosis with both asthma and AC (5%), with a final sample size of 83 (84%); see Figure S1. All subjects gave their informed consent for inclusion before they participated in the study. The study was conducted in accordance with the Declaration of Helsinki, and the protocol was approved by the Ethics Committee of Kanazawa University (Project identification code 981).

Details of guidelines and/or criteria used for diagnoses have been described elsewhere [17,18]. Patients continued to take their usual medications, according to the standard medical treatment of each disease during the study period. Patients with asthma and cough variant asthma took medications such as bronchodilator and/or ICS (inhaled corticosteriods), and patients with atopic cough took medications such as histamine H1 antagonists and/or ICS. No patient experienced symptoms suggestive of chronic obstructive pulmonary diseases or other potentially confounding cardiorespiratory disorders.

### 2.2. Health Surveys

A staged entry of participants was used (based on first consultation day during the study in which each participant was issued with cough diary). Each recorded frequency of cough in a 5 likert-scale: 1, no cough; 2, less than 5 times; 3, 6–10 times; 4, 11–20 times and 5 as over 21 times for morning, afternoon, evening and nighttime. The time period of study for each participant was from his/her first consultation day to the end of study, 30 June for all. To minimize dropout due to lack of compliance with recording, participants were asked to show their diaries to the doctor when they visited the hospital during the study period. Participants returned the diaries in person or mailed back to the hospital at the end of the study. We used the information to create a variable representing prevalence of cough symptom.

Other collected information included standard demographics, smoking status, clinical data related to asthma, cough variant asthma and atopic cough including atopy, exhaled NO.

### 2.3. Atopy

Specific Immunoglobulin E (sIgE) antibodies to 15 common aeroallergens (pollen, grass, dust mite, dog and cat dander) were measured by radioallergosorbent tests and defined atopy as having at least one sIgE > 0.35 IU/mL.

### 2.4. NO Measurement

Exhaled NO concentrations were measured by the online method using a chemiluminescence analyser (Model 280, Sievers Instruments, Boulder, CO, USA) according to the American Thoracic Society (ATS) guidelines [24]. Expiratory flow was 0.05 L/s as recommended by the guidelines and exhalation pressure was 16 cm H_2_O. Measurement of exhaled NO was repeated until three reproducible NO plateau values were achieved and the mean of these values was used as exhaled NO. Exhaled NO values were divided into two levels with a cut-off point set at 50 ppb on the basis of the recommendation by American Thoracic Society [25]. All participants had one measurement performed.

### 2.5. Ambient Air Monitoring

Daily ambient air monitoring was performed to correspond with the period participants were recording their daily symptoms. Six individual PAH compounds which included fluoranthene, pyrene, chrysene, benzo[b]fluoranthene, benzo[k]fluoranthene, and benzo[a]pyrene were measured. The sum of these individual PAH was used as proxy for total particulate PAH concentration. These were measured as part of total suspended particles (TSP) on a 24-h (noon–noon) basis from 4 January–30 June 2011 at Kanazawa University, Japan (136.7 °E, 36.6 °N). TSP was collected on boronsilicate glass fiber filter coated with fluorocarbon (FIBERFILM T60A20, 8 × 10 in, Pallflex, Putnam, CT, USA) using a high volume air sampler (120SL, KIMOTO Electric Co. Ltd., Osaka, Japan) at a flow rate of 1000 L·min^−1^.

Details of PAH extraction and quantification method has been previously described [18,26]. Hourly NO_2_, SO_2_ (for same study period) obtained from Kodatsuno monitoring site (136.7 °E, 36.6 °N) while temperature and humidity from Kanazawa Local Meteorological Office—Japan Meteorological Agency (136.6 °E, 36.6 °N) in Kanazawa city in Kanazawa city [17] were used to calculate the 24-h daily averages corresponding to PAH concentration.

### 2.6. Statistical Analysis

Analysis was performed as preplanned and restricted to only participants whose cough symptom and smoking status information had been recorded. Also, daily concentrations of pollutants of interest were available. Spearman correlation coefficients were computed to examine the association between pollutants of interest and weather parameters (temperature and humidity). Additionally, for each participant daily cough symptom was created using maximum value from afternoon to afternoon period and also average value for the entire study period calculated. The two values were then used to create a new overall day cough symptom value in order to correspond to 24-h exposure monitoring that was performed on noon-to-noon basis. The outcome variable cough prevalence (irrespective of its occurrence on the previous day) in the logistic models was defined as follows: no cough if either the difference was negative or same between the two values; cough if there was a positive difference [13]. Mann-Whitney U test was used to examine differences in the crude outcome summary (number of participants with cough symptom/number of participants who recorded for that calendar day) for entire study period between two groups. Separate logistic regression models for same day (lag0 defined as 24 h period starting from noon of calendar day before the health response) to 2-day lag as well as lag02 (average of lag0 to 2) were constructed to analyze the relationship between cough prevalence and pollutant exposure (continuous variable) on the basis of our previous analysis [18].

We used marginal approach generalized estimating equations (GEE) for panel data [17,27,28,29]. GEE models were tested using binary logistic in SPSS and first-order autoregressive correlation structure (AR1) was chosen in order to account for possible correlations between repeated measures on the same subject [27]. We also considered participant-specific intercepts to adjust for differences in cough prevalence between the participants. These were done without and with adjustment for subject-specific variables (gender, smoking status, age, disease group, BMI, atopy) and potential time dependent confounders (day of week, temperature and humidity) based on previous studies [15,17]. Temperature and humidity were treated as linear variables and were varied along with the pollutants. We also explored association on basis of two groups using (interquartile range) IQR as cut point for low and high pollutant exposure. Finally, fully adjusted multipollutant models were fitted to assess changes that occurred as a result of co-pollutant exposure.

Model regression parameters are presented as symptom odds ratios with 95 percent CI per IQR change in pollutants of interest because they are from separate measurements. All statistical analyses were performed using SPSS software program for MS Windows, version 19.0 (SPSS, Inc., New York, NY, USA) and plots made using R software (R Foundation for Statistical Computing, Vienna, Austria). Significance was set at *p* < 0.05 for all analyses.

## 3. Results

**Figure 1:**
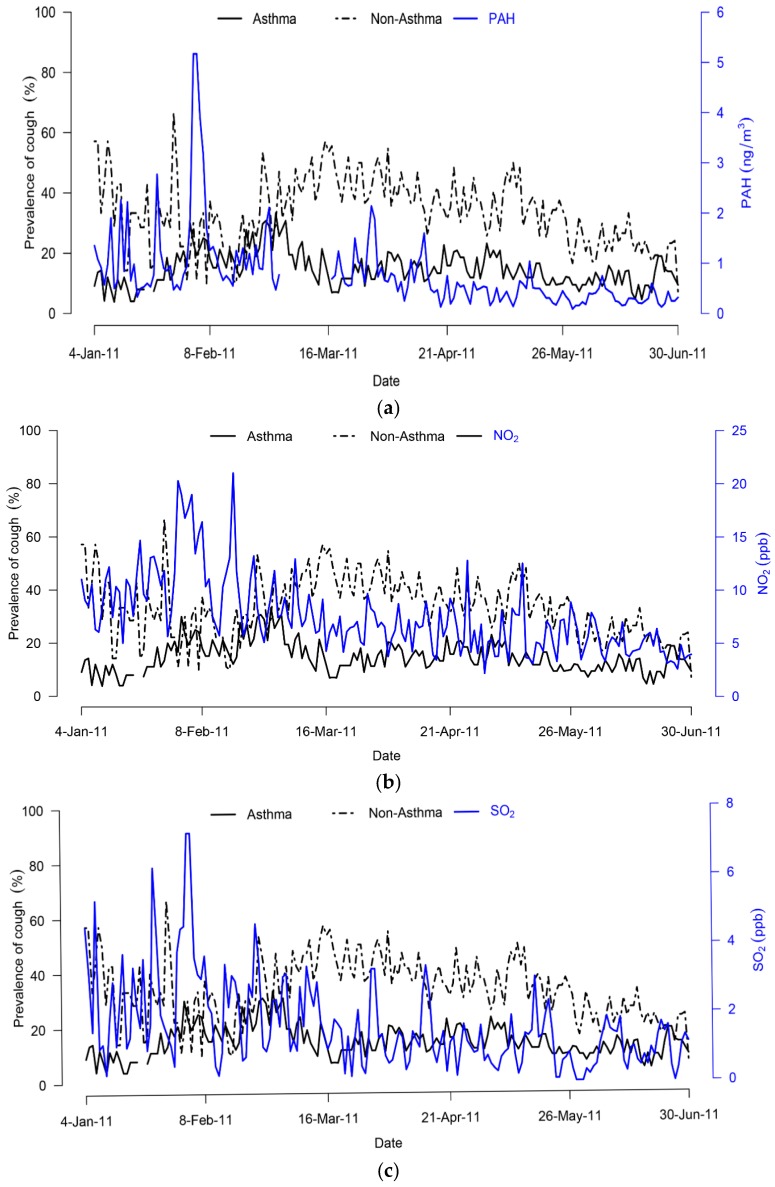
Daily time-series plots of cough prevalence for asthma and non-asthma patients (%) vs. (**a**) PAH concentrations (ng/m^3^); (**b**) NO_2_ (ppb); (**c**) SO_2_ (ppb) in Kanazawa city during 4 January–30 June 2011 study period.

Table 1 shows descriptive statistics of study participants divided into two groups according to physician diagnosis. A total of 11,913 participant-days were available for analysis from repeated measurements on 49 (asthma) and 34 (non-asthma) that were followed for 21–178 and 56–178 days for two groups respectively. Both groups had more females (over 60%) in comparison to male participants. Ten (12%) of 83 patients were diagnosed as having both CVA and AC. No significant differences were observed between age or BMI among the two groups. Except for a few days, the daily prevalence of cough was generally higher among the non-asthma group than the asthma group, with the overall prevalence for the entire study period being significantly different *p* < 0.001, (Figure 1).

Daily level of air pollutants, temperature and humidity are summarized in Table 2. For the entire study period, PAH concentrations had 8% missing data due to a technical problem with the high volume air sampler used in collection of TSP, while temperature and humidity had 0.5% missing data. Only on one day (5 February) during the entire study period was the PAH concentration measurement above 5 ng/m^3^. Both NO_2_ and SO_2_ had complete data while none of the daily averaged concentrations exceeded the WHO standards of 21.28 and 7.63 ppb, respectively. No imputation of missing data was performed. Table 3 shows Spearman’s correlation matrix of air pollutants of interest with weather parameters during the entire study period.

### 3.1. Single-Pollutant Models

Adjusted odds ratios of cough prevalence per IQR in PAH, NO_2_ and SO_2_ exposure for single pollutant models are shown in Table 3. These estimates are from regression models fitted for each pollutant as a continuous variable with adjustments (see Section 2.6). All the pollutants had robust effects for lag2 and lag02 for Asthma group with the exception of lag02 PAH exposure. Conversely, only lag2 PAH exposure had statistically significant for non-asthma group. Although in general similar trends were seen for the case of unadjusted models (see supplementary Table S1), mixed results were observed for the estimates of high versus low exposures using IQR as cut point (results presented in Table 4).

### 3.2. Multipollutant Models

Table 5 shows the adjusted estimates of cough prevalence per 0.57 ng/m^3^ PAH exposure in multipollutant models to adjust for potential confounding effects arising from copollutants. These were adjusted in addition to those made in single pollutant models (see Section 2.6). For all the pollutants, similar trends to those obtained in single pollutant models were observed except for minor changes in the coefficients and statistical significance. Results from NO_2_ and SO_2_ are represented in supplementary information (Tables S2a and S2b respectively).

## 4. Discussion

Our results displayed moderate associations between IQR increase in delayed exposure and greater odds of cough prevalence in asthma and non-asthma adult patients for the entire study period. The same associations were present after adjusting for subject-specific variables (gender, smoking status, age, disease group, BMI, atopy, exhaled NO) and potential time dependent confounders (day of week, temperature and humidity). To the best of our knowledge, this is among the few longitudinal studies to report a relationship between ambient PAH in addition to the routinely monitored pollutants and cough prevalence in asthma, CVA and/or AC patients.

While previous observational studies have assessed the relationship between ambient exposure to criteria pollutants on respiratory health among susceptible adults, there have been relatively few studies on cough symptoms associated with low level ambient PAH in addition to criteria pollutants. Thus, the associations of such exposures with cough prevalence among adults with known airway disease are not well known. A longitudinal study among susceptible (chronic obstructive pulmonary disease, asthma and ischemic heart disease) patients showed links between reduced lung function in asthmatics and NO_2_ exposure [30]. Another European multicentre panel study showed increases in previous-day coarse particle levels related to increase of respiratory symptoms [13]. Evidence of high prevalence of respiratory symptoms in adults associated with long-term exposure to air pollution of rather low levels has also been reported [31].

The current findings on PAH exposure as an independent pollutant confirm our recent result indicating that ambient particulate PAH is related to cough symptoms in adult patients with known airway disease [18]. Although differences in study population and ambient concentrations may limit direct comparisons with other findings, our results are in agreement with those studies that found increases in cough and bronchial hyperreactivity as well as high prevalence of wheezing and reduced lung function [16,32]. Another study conducted among myocardial infarction survivors reported increased odds ratio for shortness of breath symptom that was associated with 3-day lagged PAH exposure [15]. It has also been shown that average outdoor PM_10_, NO_2_ and SO_2_ was associated with reduced lung function in adults [33]. In the present study we found both groups had increases in cough prevalence with the non-asthma (CVA and AC) group seemingly influenced by ambient PAH. The same positive association remained even after controlling for concurrent exposure in two-pollutant model with signs of confounding.

We also observed NO_2_ and SO_2_ exposure were related to cough prevalence as independent pollutants among the asthma group. However, there were confounding effects when these pollutants were fitted in multipollutant models that might have resulted from moderate correlations between them. On the other hand, no significant association was found in the non-asthma group that might be due to lack of statistical power to detect the effects or different responses in asthma and non-asthma group. Previous studies on NO_2_ observed no or inconsistent associations [13,14], while some found decrement in lung function as well as increase in respiratory symptoms [31,34]. Elsewhere, a study on SO_2_ exposure reported marginal association with asthma exacerbation in children [29]. Also, adverse respiratory outcomes in adult have been reported [33,35].

Based on the current results, ambient PAH may act independently as well as synergistically with criteria pollutants (NO_2_ and SO_2_) leading to increased cough responses in adult patients with known respiratory conditions. Although low levels of ambient pollutant may have little impact on healthy persons, those with preexisting respiratory conditions might experience exaggerated responses. Among the predictor variables, disease group had a significant correlation with cough prevalence, suggesting the influence of airway disease. Chronic cough is known to result from host-environment interactions, with environmental factors such as chemicals, scents, cold air and exercise cited as common triggers of cough in chronic cough patients [11,12]. It is further suggested that increased cough responses seen in chronic cough patients could result from heightened sensitivity of cough receptors or changes in central processing or the brainstem [36,37]. Several studies have shown agonists of transient receptor potential (TRP) ion channels can evoke cough [20,38] and enhanced cough reflex resulting from increased expression of TRP ion channels has also been reported [39]. In addition, a report on transient receptor potential vanilloid 1 (TRPV1) polymorphisms associated with cough in subjects without asthma has been published [40] while recent studies have further shown TRPA1 as a promiscuous receptor for a wide range of stimuli [19,38]. It may be presumed that activation of these receptors following exposure to environmental irritants might have led to the increased cough responses as observed in this study. However, we can not rule out involvement of other pathways and further biological studies on mechanisms of cough sensitivity between these two groups might help to better explain the outcomes resulting from such exposure.

The strength of this study emanates from use of daily concentrations of ambient PAH (sum of six individual PAH) that allowed evaluation of the relationship between organic compounds not routinely monitored with cough symptoms alongside NO_2_ and SO_2_. Additionally, all the participants in the current study were physician-diagnosed to at least have a clinical condition associated with chronic cough that provided an opportunity to study a sub-group of the population at risk. Furthermore, repeated measurements on the same panel of participants had the advantage of detecting associations between health outcome and subtle changes in ambient exposures with each one of them acting as their own control [15].

Some study limitations need to be considered. First, the small sample size of non-asthma group consisting of some diagnosed with both CVA and AC might have reduced the statistical power, though an association was found in relation to PAH exposure. Second, there was the masking of adverse effects due to the fact that participants continued to take their prescribed medication during the study. However, no changes in prescription of medication were effected that could be interpreted, as no significant influence of medication use was present in the current study. Third, daily pollutants were collected from central sites, and hence the possibility of misclassification of exposure especially for participants who resided at greater distances from the central monitoring sites. However, in the present study ambient exposures irrespective of source were of interest.

## 5. Conclusions

In conclusion, our results suggest that ambient PAH, NO_2_, SO_2_ is associated with increases in cough prevalence among adult chronic cough patients, with the largest associations seen in lag2 and lag02 exposures. Between the two groups, non-asthma patients are more likely to have increased cough prevalence that is related to ambient PAH exposure, while asthma patients may experience increases related to ambient pollutants in general. Our results add to the growing evidence that ambient pollutants, including organic chemical compounds not routinely monitored even at low levels, remain a matter of concern that needs to be considered as aggravating factors in adult patients with chronic cough.

## Figures and Tables

**Table 1:** Descriptive statistics of study participants for study period 4 January–30 June 2011, Kanazawa city **^a^**.

Subject Characteristic	Asthma, *n* = 49	Non-Asthma, *n* = 34
Median age (age range, years)	67 (23–84)	62 (29–79)
Gender		
Males (%)	18 (37)	10 (29)
Females (%)	31 (63)	24 (71)
BMI (SD, Kg/m^2^)	22.21 (2.60)	23.16 (4.10)
Disease		
Asthma (%)	49 (100)	
CVA (%)		8 (24)
AC (%)		16 (47)
CVA and AC (%)		10 (29)
Smoking status		
Never smoked (%)	29 (59)	29 (85)
Ex-smoker (%)	20 (41)	5 (15)
Number of recorded days	141.2	121.5
Cough prevalence (%) **^b^**	14.8	33.8
Time period of study (days) **^c^**	152.5	131.8
Atopy(%) **^d^**	28 (57)	15 (44)
Exhaled NO (%) **^e^**	12 (22)	1 (3)

Abbreviation: SD, standard deviation; CVA, cough variant asthma; AC, atopic cough; NO, nitric oxide; asthma (asthma only); non-asthma (AC, CVA, CVA and AC). **^a^** study participants after excluding 5 current smokers, 3 with no smoking status, 3 with no cough symptom information, 5 diagnosed with asthma and AC; **^b^** number of participants with cough symptom/number of participants who recorded for that calendar day, Mann-Whitney U test for difference in cough prevalence between asthma and non-asthma group, *p* < 0.001; **^c^** from entry (first consultation day during study period for each participant) to the end of study period (30 June for all); **^d^** number of participants with at least one sIgE ≥ 0.35 IU/mL; **^e^** number of participants with Exhaled NO value >50 ppb on basis of recommendations by American Thoracic Society.

**Table 2:** Summary of air pollutants, temperature and humidity for the entire study period and Spearman’s correlations, 4 January–30 June 2011, Kanazawa city **^a^**.

Variable	PAH (ng/m^3^) ^b^	NO_2_ (ppb) ^c^	SO_2_ (ppb) ^c^	Temperature (°C) ^d^	Humidity (%) ^d^
Minimum	0.09	2.17	0.00	−1.8	36
Maximum	5.17	21.00	7.29	28.6	91
Mean (SD)	0.75 (0.67)	7.49 (3.50)	1.60 (1.25)	10.7 (8.05)	68.36 (11.37)
IQR	0.57	4.08	1.47	14.4	16.0
Correlations					
PAH	1	0.508 **^†^**	0.598 **^†^**	−0.635 **^†^**	−0.332 **^†^**
NO_2_		1	0.560 **^†^**	−0.617 **^†^**	−0.121
SO_2_			1	−0.357 **^†^**	−0.242 **^†^**
Temperature				1	−0.102
Humidity					1

Abbreviations: PAH, polycyclic aromatic hydrocarbon; PAH includes fluoranthene, pyrene, chrysene, benzo[b]fluoranthene, benzo[k]fluoranthene, benzo[a]pyrene; NO_2_, nitrogen dioxide; SO_2_, sulphur dioxide; SD, standard deviation; IQR, interquartile range. **^a^** Measurements used were from 3 central monitoring sites; **^b^** Measurements were taken at Kanazawa University site and 15 days are missing due to equipment failure (*n* = 163 days); **^c^** Data obtained from Kodatsuno site (*n* = 178 days); **^d^** Data obtained from Kanazawa Local Meteorological Office, Japan Meteorological Agency with 1 day missing data (*n* = 177); **^†^** *p* < 0.001.

**Table 3:** Adjusted odds ratios for cough prevalence per IQR change in pollutants (as continuous variable) in single pollutant model (4 January–30 June 2011) **^a^**.

Pollutant		Asthma, *n* = 49		Non-Asthma, *n* = 34	
		OR	95% CI	OR	95% CI
PAH	Lag0	0.988	0.935, 1.044	1.024	0.959, 1.093
	Lag1	0.998	0.926, 1.075	0.964	0.893, 1.041
	Lag2	**1.083**	**1.029, 1.140**	**1.097**	**1.016, 1.185**
	Lag02	1.057	0.975, 1.146	0.986	0.885, 1.098
NO_2_	Lag0	0.964	0.879, 1.057	0.980	0.880, 1.091
	Lag1	1.065	0.977, 1.160	1.069	0.944, 1.210
	Lag2	**1.093**	**1.005, 1.188**	1.092	0.984, 1.211
	Lag02	**1.161**	**1.049, 1.286**	1.087	0.873, 1.352
SO_2_	Lag0	0.942	0.852, 1.041	0.991	0.900, 1.091
	Lag1	1.033	0.953, 1.119	1.085	0.961, 1.225
	Lag2	**1.122**	**1.044, 1.204**	1.071	0.940, 1.221
	Lag02	**1.146**	**1.007, 1.304**	1.116	0.967, 1.288

Abbreviations: OR, odds ratio; CI, confidence interval; PAHs, polycyclic aromatic hydrocarbons, PAHs includes fluoranthene, pyrene, chrysene, benzo[b]fluoranthene, benzo[k]fluoranthene, benzo[a]pyrene; NO_2_, nitrogen dioxide; SO_2_, sulphur dioxide. **^a^** values in bold are statistically significant (*p* < 0.05); adjusted for age, gender, BMI, atopy, smoking status, exhaled NO, disease group, day of week, temperature, humidity. Estimates are per values of IQR as in Table 2.

**Table 4:** Adjusted odds ratios for cough prevalence per IQR change for high and low level pollutant exposure (4 January–30 June, 2011) **^a^**.

Pollutant		Exposure	Asthma, *n* = 49		Non-Asthma, *n* = 34	
			OR	95% CI	OR	95% CI
PAH	Lag0	High	0.966	0.882, 1.058	1.040	0.936, 1.155
		Low	1		1	
	Lag1	High	1.019	0.921, 1.129	0.962	0.893, 1.036
		Low	1		1	
	Lag2	High	1.087	0.998, 1.184	**1.120**	**1.028, 1.219**
		Low	1		1	
	Lag02	High	1.086	0.950, 1.243	1.068	0.987, 1.156
		Low	1		1	
NO_2_	Lag0	High	0.625	0.296, 1.317	0.699	0.374, 1.307
		Low	1		1	
	Lag1	High	0.940	0.477, 1.853	1.548	0.902, 2.658
		Low	1		1	
	Lag2	High	1.527	0.917, 2.542	1.543	0.842, 2.825
		Low	1		1	
	Lag02	High	1.112	0.459, 2.694	1.415	0.736, 2.721
		Low	1		1	
SO_2_	Lag0	High	0.995	0.809, 1.224	0.983	0.808, 1.194
		Low	1		1	
	Lag1	High	0.924	0.730, 1.169	**1.255**	**1.077, 1.462**
		Low	1		1	
	Lag2	High	**1.215**	**1.019, 1.447**	0.999	0.791, 1.260
		Low	1		1	
	Lag02	High	1.008	0.782, 1.301	**1.286**	**1.064, 1.553**
		Low	1		1	

Abbreviations: IQR, interquartile range; OR, odds ratio; CI, confidence interval; PAH, polycyclic aromatic hydrocarbon, includes fluoranthene, pyrene, chrysene, benzo[b]fluoranthene, benzo[k]fluoranthene, benzo[a]pyrene; NO_2_, nitrogen dioxide; SO_2_, sulphur dioxide. **^a^** values in bold are statistically significant (*p* < 0.05) and low pollutant level set as reference category. Adjusted for age, gender, BMI, atopy, smoking status, exhaled NO, disease group, day of week, temperature, humidity. Estimates are per values of IQR as in Table 2.

**Table 5:** Adjusted odds ratios for cough prevalence per IQR change in PAH exposure in multipollutant model (4 January–30 June 2011) **^a^**.

Pollutant		Asthma, *n* = 49		Non-Asthma, *n* = 34	
		OR	95% CI	OR	95% CI
adjusted NO_2_	Lag0	0.988	0.932, 1.046	1.031	0.963, 1.103
	Lag1	0.989	0.918, 1.066	0.954	0.882, 1.031
	Lag2	**1.068**	**1.018, 1.119**	**1.092**	**1.009, 1.183**
	Lag02	1.024	0.946, 1.108	0.969	0.865, 1.085
adjusted SO_2_	Lag0	1.004	0.943, 1.069	1.037	0.954, 1.127
	Lag1	0.981	0.905, 1.064	0.937	0.863, 1.018
	Lag2	**1.056**	**1.010, 1.104**	**1.087**	**1.010, 1.169**
	Lag02	0.999	0.921, 1.084	0.917	0.823, 1.023

Abbreviations: IQR, interquartile range; OR, odds ratio; CI, confidence interval; PAH, polycyclic aromatichydrocarbons, includes fluoranthene, pyrene, chrysene, benzo[b]fluoranthene, benzo[k]fluoranthene, benzo[a]pyrene; NO_2_, nitrogen dioxide; SO_2_, sulphur dioxide. **^a^** values in bold are statistically significant (*p* < 0.05), Adjusted for NO_2_ and SO_2_ in addition to age, gender, BMI, atopy, smoking status, exhaled NO, disease group, day of week, temperature, humidity. Estimates are per values of IQR as in Table 2.

## Supplementary Materials

The following are available online at www.mdpi.com/1660-4601/13/8/800/s1, Figure S1: Flowchart of study participants and exclusions., Table S1: Unadjusted odds ratios of cough prevalence per IQR change in pollutants (as continuous variable) in single pollutant model (4 January–30 June 2011), Table S2a: Adjusted odds ratios for cough prevalence per IQR change in NO_2_ exposure in multipollutant model (4 January–30 June 2011), Table S2b: Adjusted odds ratios for cough prevalence per IQR change in SO_2_ exposure in multipollutant model (4 January–30 June 2011).

## Abbreviations

The following abbreviations are used in this manuscript:

PAH	polycyclic aromatic hydrocarbons
DEP	diesel exhaust particles
PM_2.5_	particulate matter less than 2.5 µm in aerodynamic diameter
PM_10_	particulate matter less than 10 µm in aerodynamic diameter
TRPA1	transient receptor potential Ankyrin 1
CVA	cough variant asthma
AC	atopic cough
NO_2_	nitrogen dioxide
SO_2_	sulphur dioxide
ICS	inhaled corticosteroids
NO	nitric oxide
sIgE	specific immunoglobulin E
TSP	total suspended particles
HPLC	high performance liquid chromatography
GEE	generalized estimating equation
IQR	interquartile range
lag0	24-h period starting from noon of calendar before health response
lag1	1 day preceding this day
lag2	2 days preceding this day
lag02	average of lag0 to 2
CI	confidence interval
OR	odds ratio
TRP	transient receptor potential
TRPV1	transient receptor potential Vanilloid 1
