# Supplementary material for: Associations of Cough Prevalence with Ambient Polycyclic Aromatic Hydrocarbons, Nitrogen and Sulphur Dioxide: A Longitudinal Study
Source: Int J Environ Res Public Health. 2016 Aug 9;13(8):800. doi: 10.3390/ijerph13080800 (PMC4997486; doi:10.3390/ijerph13080800)
Supplement: Supplementary file 1 [file ijerph-13-00800-s001.pdf]

Supplementary Materials: Associations of Cough Prevalence with Ambient Polycyclic Aromatic Hydrocarbons, Nitrogen and Sulphur Dioxide: A Longitudinal Study

Enoch Olando Anyenda, Tomomi Higashi, Yasuhiro Kambayashi, Thao Thi Thu Nguyen, Yoshimasa Michigami, Masaki Fujimura, Johsuke Hara, Hiromasa Tsujiguchi, Masami Kitaoka, Hiroki Asakura, Daisuke Hori, Yohei Yamada, Koichiro Hayashi, Kazuichi Hayakawa and Hiroyuki Nakamura

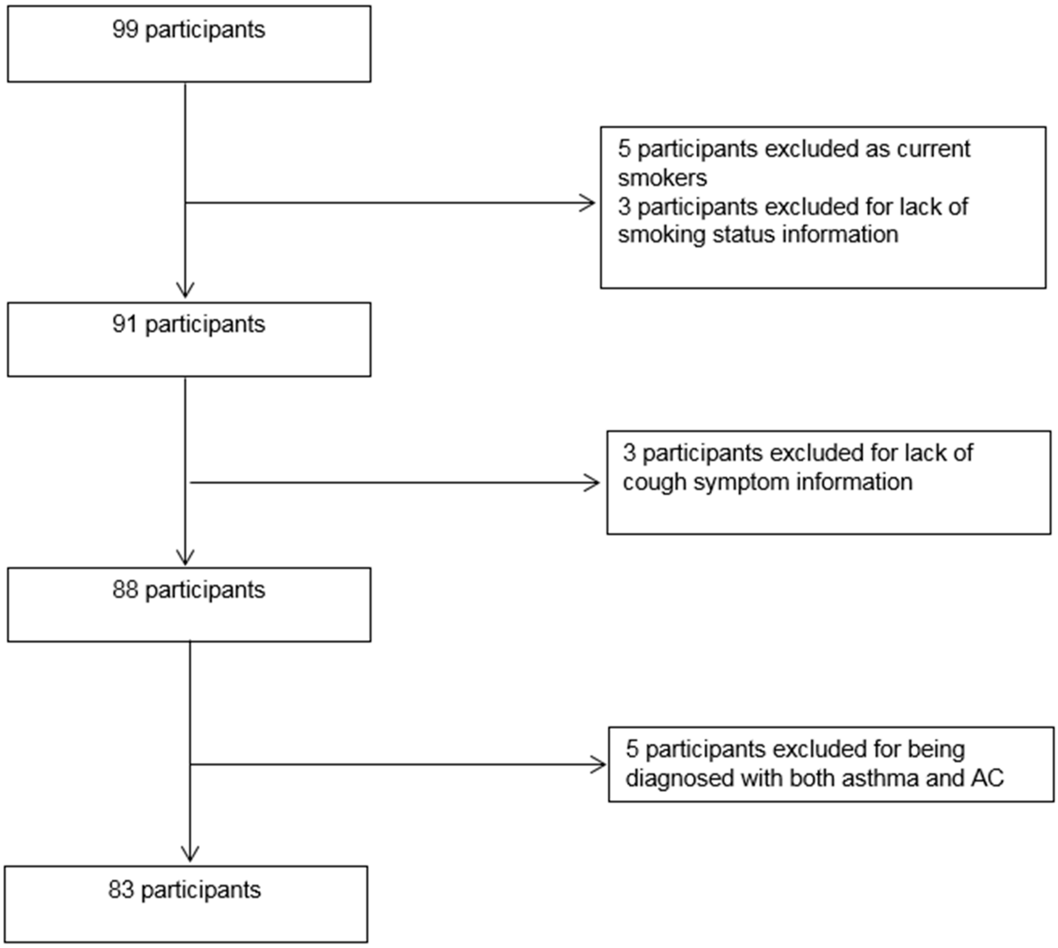

Figure S1. Flowchart of study participants and exclusions.

Table S1. Unadjusted odds ratios of cough prevalence per IQR change in pollutants (as continuous variable) in single pollutant model (4 January–30 June 2011) [a].

Pollutant		Asthma, *n* = 49		Non-Asthma, *n* = 34	
		OR	95% CI	OR	95% CI
PAH	Lag0	0.997	0.949, 1.048	1.029	0.962, 1.101
	Lag1	0.988	0.912, 1.071	0.960	0.896, 1.029
	Lag2	**1.067**	**1.019, 1.117**	**1.093**	**1.026, 1.165**
	Lag02	1.050	0.974, 1.133	1.054	0.955, 1.163
NO_2	Lag0	0.996	0.914, 1.085	1.037	0.935, 1.150
	Lag1	1.034	0.954, 1.121	1.057	0.973, 1.149
	Lag2	1.050	0.965, 1.141	0.990	0.913, 1.074
	Lag02	**1.097**	**1.001, 1.202**	1.054	0.903, 1.229
SO_2	Lag0	0.964	0.871, 1.066	1.002	0.932, 1.076
	Lag1	1.011	0.934, 1.096	1.063	0.965, 1.172
	Lag2	**1.077**	**1.012, 1.147**	1.042	0.948, 1.146
	Lag02	1.102	0.988, 1.230	1.107	0.991, 1.237

Abbreviations: OR, odds ratio; CI, confidence interval; PAHs, polycyclic aromatic hydrocarbons, PAHs includes fluoranthene, pyrene, chrysene, benzo[b]fluoranthene, benzo[k]fluoranthene, benzo[a]pyrene; NO_2, nitrogen dioxide; SO_2, sulphur dioxide. [a] values in bold are statistically significant ($p < 0.05$); adjusted for age, gender, BMI, atopy, smoking status, exhaled NO, disease group, day of week, temperature, humidity. Estimates are per values of IQR as in Table 2.

Table S2a. Adjusted odds ratios for cough prevalence per IQR change in NO_2 exposure in multipollutant model (4 January–30 June 2011) [a].

Pollutant		Asthma, *n* = 49		Non-Asthma, *n* = 34	
		OR	95% CI	OR	95% CI
adjusted PAH	Lag0	1.001	0.899, 1.115	0.947	0.841, 1.067
	Lag1	1.085	0.982, 1.199	1.097	0.966, 1.245
	Lag2	**1.123**	**1.025, 1.231**	1.029	0.918, 1.152
	Lag02	**1.198**	**1.070, 1.343**	1.096	0.863, 1.392
adjusted SO_2	Lag0	0.983	0.890, 1.086	0.981	0.867, 1.110
	Lag1	1.059	0.959, 1.169	1.047	0.901, 1.216
	Lag2	1.057	0.965, 1.158	1.077	0.961, 1.207
	Lag02	1.106	0.971, 1.261	1.039	0.819, 1.319

Abbreviations: IQR, interquartile range; OR, odds ratio; CI, confidence interval; PAH, polycyclic aromatichydrocarbons, includes fluoranthene, pyrene, chrysene, benzo[b]fluoranthene, benzo[k]fluoranthene,benzo[a]pyrene; NO_2, nitrogen dioxide;SO_2, sulphur dioxide. [a] values in bold are statistically significant ($p < 0.05$), Adjusted for PAH and SO_2 in addition to age, gender, BMI, atopy, smoking status, exhaled NO, disease group, day of week, temperature, humidity. Estimates are per values of IQR as in Table 2.

Table S2b. Adjusted odds ratios for cough prevalence per IQR change in SO_2 exposure in multipollutant model (4 January–30 June 2011) [a].

**Pollutant**		**Asthma, *n* = 49**		**Non-Asthma, *n* = 34**	
		**OR**	**95% CI**	**OR**	**95% CI**
adjusted PAH	Lag0	0.939	0.841, 1.098	0.959	0.838, 1.098
	Lag1	1.068	0.977, 1.287	1.108	0.954, 1.287
	Lag2	**1.106**	**1.040, 1.178**	1.034	0.907, 1.178
	Lag02	**1.183**	**1.032, 1.438**	1.205	1.009, 1.438
adjusted NO_2	Lag0	0.947	0.850, 1.116	0.997	0.890, 1.116
	Lag1	1.016	0.926, 1.241	1.071	0.924, 1.241
	Lag2	**1.102**	**1.022, 1.206**	1.048	0.910, 1.206
	Lag02	1.096	0.938, 1.284	1.100	0.942, 1.284

Abbreviations: IQR, interquartile range; OR, odds ratio; CI, confidence interval; PAH, polycyclic aromatichydrocarbons, includes fluoranthene, pyrene, chrysene, benzo[b]fluoranthene, benzo[k]fluoranthene, benzo[a]pyrene; NO_2, nitrogen dioxide; SO_2, sulphur dioxide. [a] values in bold are statistically significant ($p < 0.05$), Adjusted for PAH and NO_2 in addition to age, gender, BMI, atopy, smoking status, exhaled NO, disease group, day of week, temperature, humidity. Estimates are per values of IQR as in Table 2.

© 2016 by the authors; licensee MDPI, Basel, Switzerland. This article is an open access article distributed under the terms and conditions of the Creative Commons by Attribution (CC-BY) license (http://creativecommons.org/licenses/by/4.0/).
